# Experimental nerve transfer model in the rat forelimb

**DOI:** 10.1007/s10353-016-0386-4

**Published:** 2016-02-01

**Authors:** K. D. Bergmeister, M. Aman, O. Riedl, K. Manzano-Szalai, M. E. Sporer, S. Salminger, O. C. Aszmann

**Affiliations:** 1 CD Laboratory for the Restoration of Extremity Function, Department of Surgery, Medical University of Vienna, Vienna, Austria; 2 Division of Biomedical Research, Medical University of Vienna, Vienna, Austria; 3 Christian Doppler Laboratory for Restoration of Extremity Function, Division of Plastic and Reconstructive Surgery, Medical University of Vienna, Spitalgasse 23, 1090 Vienna, Austria

**Keywords:** Nerve transfers, Extremity reconstruction, Rat nerve transfer model, Targeted muscle reinnervation, Peripheral nerve surgery

## Abstract

**Background:**

Nerve transfers are a powerful tool in extremity reconstruction, but the neurophysiological effects have not been adequately investigated. As 81 % of nerve injuries and most nerve transfers occur in the upper extremity with its own neurophysiological properties, the standard rat hindlimb model may not be optimal in this paradigm. Here we present an experimental rat forelimb model to investigate nerve transfers.

**Methods:**

In ten male Sprague-Dawley rats, the ulnar nerve was transferred to the motor branch of long head of the biceps. Sham surgery was performed in five animals (exposure/closure). After 12 weeks of regeneration, muscle force and Bertelli test were performed and evaluated.

**Results:**

The nerve transfer successfully reinnervated the long head of the biceps in all animals, as indicated by muscle force and behavioral outcome. No aberrant reinnervation occurred from the original motor source. Muscle force was 2,68 N ± 0.35 for the nerve transfer group and 2,85 N ± 0.39 for the sham group, which was not statically different (*p* = 0.436). The procedure led to minor functional deficits due to the loss of ulnar nerve function; this, however, could not be quantified with any of the presented measures.

**Conclusion:**

The above-described rat model demonstrated a constant anatomy, suitable for nerve transfers that are accessible to standard neuromuscular analyses and behavioral testing. This model allows the study of both neurophysiologic properties and cognitive motor function after nerve transfers in the upper extremity.

## Introduction

Nerve transfers have been used in the past decades by reconstructive surgeons to treat upper extremity injuries [1–4]. In this application, peripheral nerves are transferred to new target muscles to either restore muscle function following nerve damages [4, 5], restore hand function in tetraplegia, or to improve the control of myoelectric prostheses by targeted muscle reinnervation (TMR) [6–8]. Clinical studies have shown that nerve transfers provide exceptional outcomes in terms of regeneration [9, 10], but limited quantities of donor nerves sometimes prevent better overall extremity function [11]. As little is known about the neurophysiological effects of nerve transfers, detailed experimental investigations may identify possibilities to refine this treatment. Currently, the standard neuromuscular experimental model is the rat hindlimb, which provides a large anatomy and many available studies on methodology or techniques [12–14]. However, 81 % of all nerve damages in humans occur in the upper extremity [15] and significant anatomical and physiological differences exist compared with the lower extremity. The same applies to nerve transfers, which are almost exclusively used in the upper extremity to restore critical hand function but rarely in the lower extremity. Important for functional outcomes is the application of nerve transfers that take synergistic function and axon ratio between donor and recipient into account. Correct axon ratio is crucial for the successful reinnervation of a high number of muscle fibers and thus muscle functionality [16]. The synergistic function between donor and recipient facilitates cortical recognition of the new innervation pattern by the patient [17]. This process of reintegrating lost muscle function via new nerve connections is in any case demanding but highly complicated if donor nerves are transferred to antagonistic muscles [5, 18].

To investigate this complex paradigm, we believe it is necessary to study nerve regeneration in a rat forelimb (upper extremity) model that closely resembles the anatomy and physiology of the upper extremity. As nerve transfers in the upper extremity need to make sense in the cognitive context, a behavioral end point analysis is especially useful. The aim of this study was thus to provide a nerve transfer model that lends it to standard neuromuscular analyses and behavioral testing. For this purpose, we specifically designed a rat nerve transfer/TMR model identical to clinical nerve transfers, where the ulnar nerve was transferred onto the long head of the biceps.

## Methods

### Experimental design

Dissections were conducted on eight euthanized animals to study the neuromuscular anatomy of the rat forelimb in comparison to human anatomy and the rat’s lower extremity. Nerve transfers were conducted in a total of ten Sprague-Dawley rats (male, aged 8–10 weeks, 350–400 g). For control, five animals received sham surgery with skin incision, exposure of the relevant nerves, and closure but no nerve transfer. The animals were treated in line with the principles of laboratory animal care as recommended by Federation of European Laboratory Animal Science Associations (FELASA) [19]. Approval was obtained prior to the study from the ethics committee of the Medical University of Vienna and the Austrian Ministry for Research and Science (BMWF: Bundesministerium fuer Wissenschaft und Forschung, reference number: BMWF-66.009/0222-WF/II/3b/2014 & BMWF-66.009/0187-WF/V/3b/2015).

### Operation: Nerve transfer

**Fig. 1:**
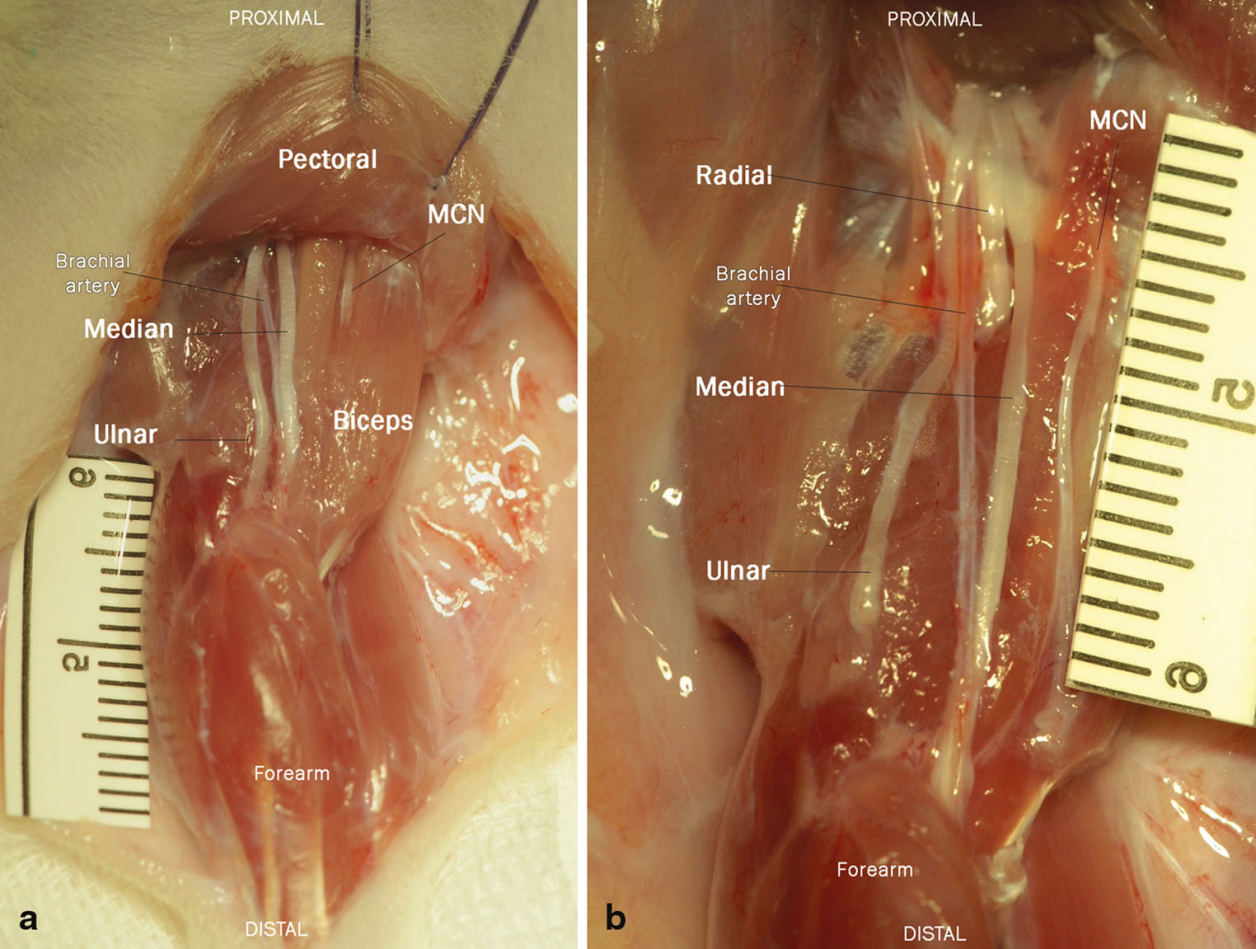
Nerve transfer anatomy. **a** Operation situs **before the nerve transfer: The ulnar donor nerve is transferred to the long head of the** biceps, and its motor branch deriving from the musculocutaneus (MCN) nerve was resected to prevent aberrant reinnervation. Clinically, identical nerve transfers are used for targeted muscle reinnervation to improve prosthetic control in amputees. **b** Nerve transfer after 12 weeks. The nerve has successfully reinnervated the long head of the biceps. (Postmortem images with pectoral muscle removed.)

A Z-shaped incision was made on the upper extremity from the pectoral muscle to the medial epicondyle of the humerus. Following blunt mobilization and preparation of the subcutaneous tissue, the skin incision was retracted with four single knot sutures to expose the neuromuscular structures of the forelimb. Under a Zeiss microscope (Munich, Germany) the venous ramus recurrens between the brachial and cephalic vein was electrocuted and divided to expose the underlying biceps. In a next step, the ulnar nerve was followed to the medial epicondyle. Due to the nerve’s proximity to the brachial vein the careful dissection was critical to prevent bleeding. Then the pectoral muscle was retracted, and the cut ulnar nerve freed from distal to proximal from the surrounding tissue. The nerve was mobilized to its origin from the medial cord of the brachial plexus to gain sufficient length for the nerve transfer. In between the biceps’ two origins, the musculocutaneus nerve was followed distally to the motor branch of the biceps’ long head. The long head of the biceps was denervated by resection of the motor branch to its origin from the musculocutaneus nerve to prevent any aberrant regeneration. In a final step, the ulnar nerve was neurotized to the epimysium of the motor branch’s insertion point via two 11–0 sutures (Ethilon, Ethicon, Johnson and Johnson Medical Care, Austria) (Fig. 1a). Using this neurotization procedure, the regeneration distance and thus the effect of denervation was kept to a minimum. Wound closure was done with 6–0 vicryl subcutaneous continuous sutures and 4–0 vicryl single-knot skin sutures. Postoperatively, all animals were controlled daily by an animal technician to document any motor or sensory deficit (developing of ulcers) and impairments in daily chores.

### Anesthesia, postoperative care, and euthanasia

Interventions were performed under general anesthesia/analgesia using ketamine (100 mg/kg)/xylazin (5 mg/kg) i.p., inhalative isoflurane (tracheal tube) and piritramide injections (piritramide 0.3 mg/kg s.c.). For pain relief on the first 5 postoperative days, the drinking water was mixed with piritramide and glucose (two ampules Dipidolor equaling 30 mg piritramide +10 mL 10 % glucose solution in 250 mL drinking water). Euthanasia was conducted under general anesthesia as described above with a 1-ml intracardial injection of pentobarbital after control of adequate anesthesia depth.

### Behavioral assessments

Global upper extremity function was analyzed with the Bertelli test [20], which was started 2 weeks after the primary surgery to allow postoperative recovery. The voluntary, bilateral grooming response following squirts with sweetened water onto the animals’ snouts was analyzed to allow functional analysis of elbow flexion and abduction. The operated forelimb was thereby compared with the contralateral healthy forelimb and subsequently graded employing the following score: grade 1 (no movement or mouth), grade 2 (region below the eye), grade 3 (eye), grade 4 (front of ears), and grade 5 (behind the ears).

### Functional Assessment

Twelve weeks after the primary surgery, muscle force analysis was used to quantify muscular reinnervation. The ulnar nerve was exposed via an incision at the medial aspect of the upper foreleg, extending from the pectoralis muscle to the median antecubital fossa. The proximal tendon of the bicep’s long head was identified, divided, and folded into a tendon loop and attached to a force transducer (BG-1000; Kulite Semiconductor Products, Leonia, NJ, USA) with one 4–0 vicryl suture. During the whole procedure, the muscle and the ulnar nerve were regularly bathed with warm saline (36 °C) in order to keep functionality intact. The rat was placed in a supine position on a platform with the humeral head and elbow firmly secured. A shielded bipolar silver wire electrode was then used to apply supramaximal stimuli (square pulses, 0.2 ms pulse duration, 2–6 V) generated by a Grass S88 Stimulator (Grass Instrument Co., Quincy, MA, USA). These were delivered to the ulnar nerve with a hook electrode in order to indirectly activate the long head. Twitch contractions were then utilized to determine optimal muscle length for force production. This length was subsequently used for all measurements. For maximum isometric tetanic force measurement, the muscle was stimulated for 250 ms at increasing frequencies from 30 to 300 Hz. To allow muscle recovery the procedure was paused for 2 min after each contraction.

### Statistical Analysis

All histological data were reported as mean values ± standard deviation. The data were visually evaluated for deviations from normality using Q plots and analyzed with a Kolmogorov–Smirnov test, which indicated a normal distribution. Statistical evaluation was then done using a two-sided Student’s *t* test in SPSS to compare means of muscle force. *p* values smaller than 0.05 were considered statistically significant. All statistical evaluations were done in SPSS (V.21, IBM Corp., USA).

## Results

### Neuromuscular anatomy

**Fig. 2:**
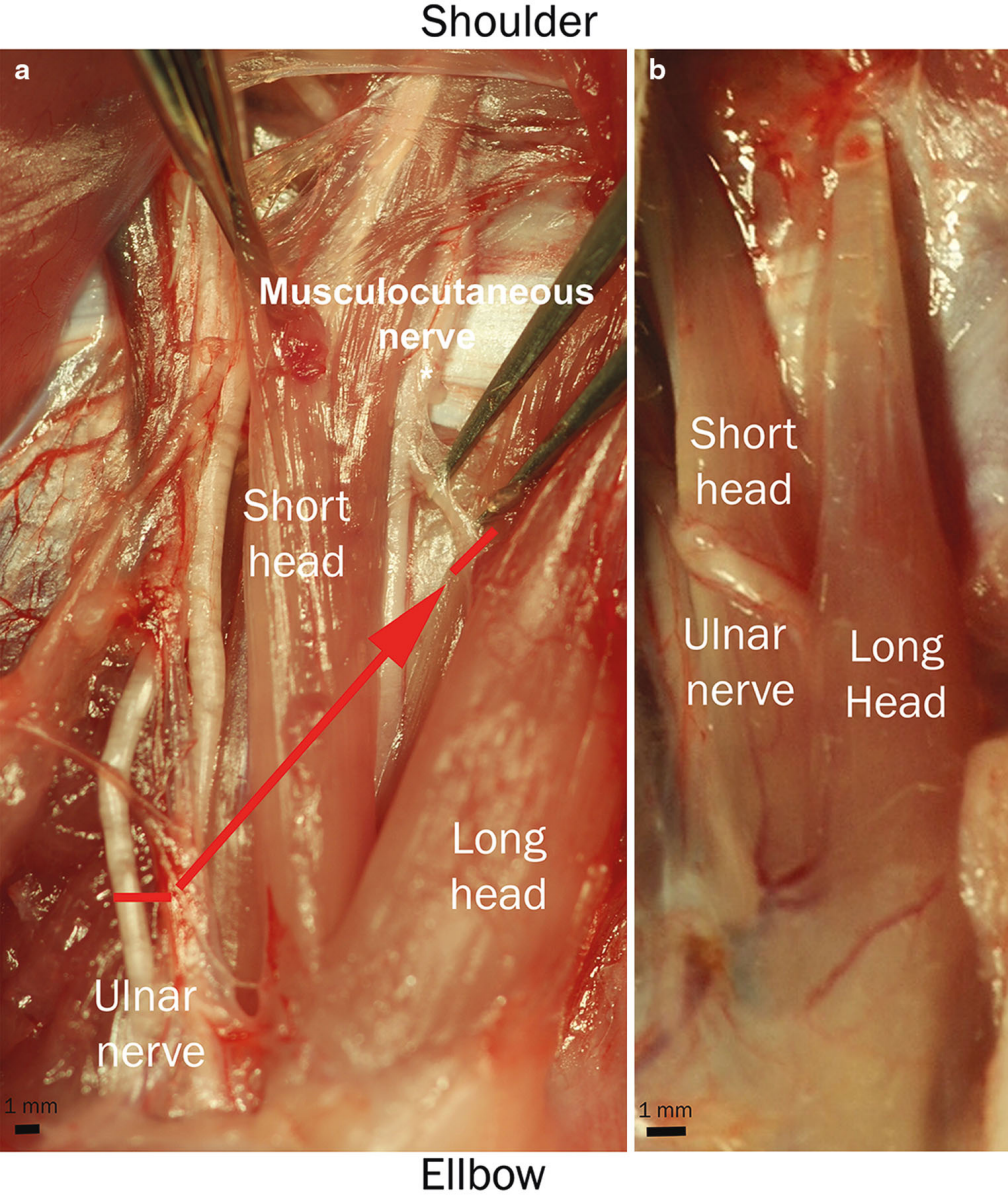
Dissections on the anatomy of the rat plexus from the anterior aspect. **a** Forelimb situs after skin incision and retracted pectoral muscle, the major nerves and muscles are well accessible. The musculocutaneus is not visible as it is concealed by the biceps. **b** Proximal situs of brachial plexus with removed biceps, visualizing the musculocutaneus nerve. The radial nerve is visible as it moves to the posterior aspect of the extremity. Notice the close proximity of the nerves to the brachial artery

The brachial plexus was composed of anatomically identical nerve structures, with the ulnar, median, and musculocutaneus nerves being most accessible for surgical manipulation (Fig. 2). The ulnar nerve presented itself as the best option for surgical manipulation due to its easy ventral accessibility. Additionally, the long constant course of the ulnar nerve originating from the medial cord of the brachial plexus and running anteromedially to the brachial artery all the way to the medial epicondyle provides sufficient length for manipulation. Its innervation pattern is similar to humans, yet its function is not critical in any motion tasks. Therefore, the ulnar nerve was chosen as a donor nerve to the long head of the biceps, which closely resembles standard TMR transfer matrices and is similar to biological nerve transfers for elbow reanimation [10, 21, 22].

### Nerve transfer surgery

Twelve weeks after the initial surgery, all donor nerves had reinnervated the target muscles (Fig. 1b). All nerve transfers were functional, indicated by muscle force analysis or neurotomy reaction during muscle harvest. Detailed inspection during the dissection and functional testing indicated that all transferred nerves successfully reinnervated the long head of the biceps and that no additional reinnervation occurred from the musculocutaneus nerve. Postoperatively, animals showed no signs of pain or distress. The nerve transfer did not affect the use of the forelimb during daily activities, and visually all animals had a normal gait even after the pain medication was withdrawn. A small ulcer on the forelimb’s fifth digit was present in only one animal due to the sensory denervation of the ulnar nerve. Mean time between skin incision and closure was 56.3 ± 17.07 min.

### Functional muscle testing

**Fig. 3:**
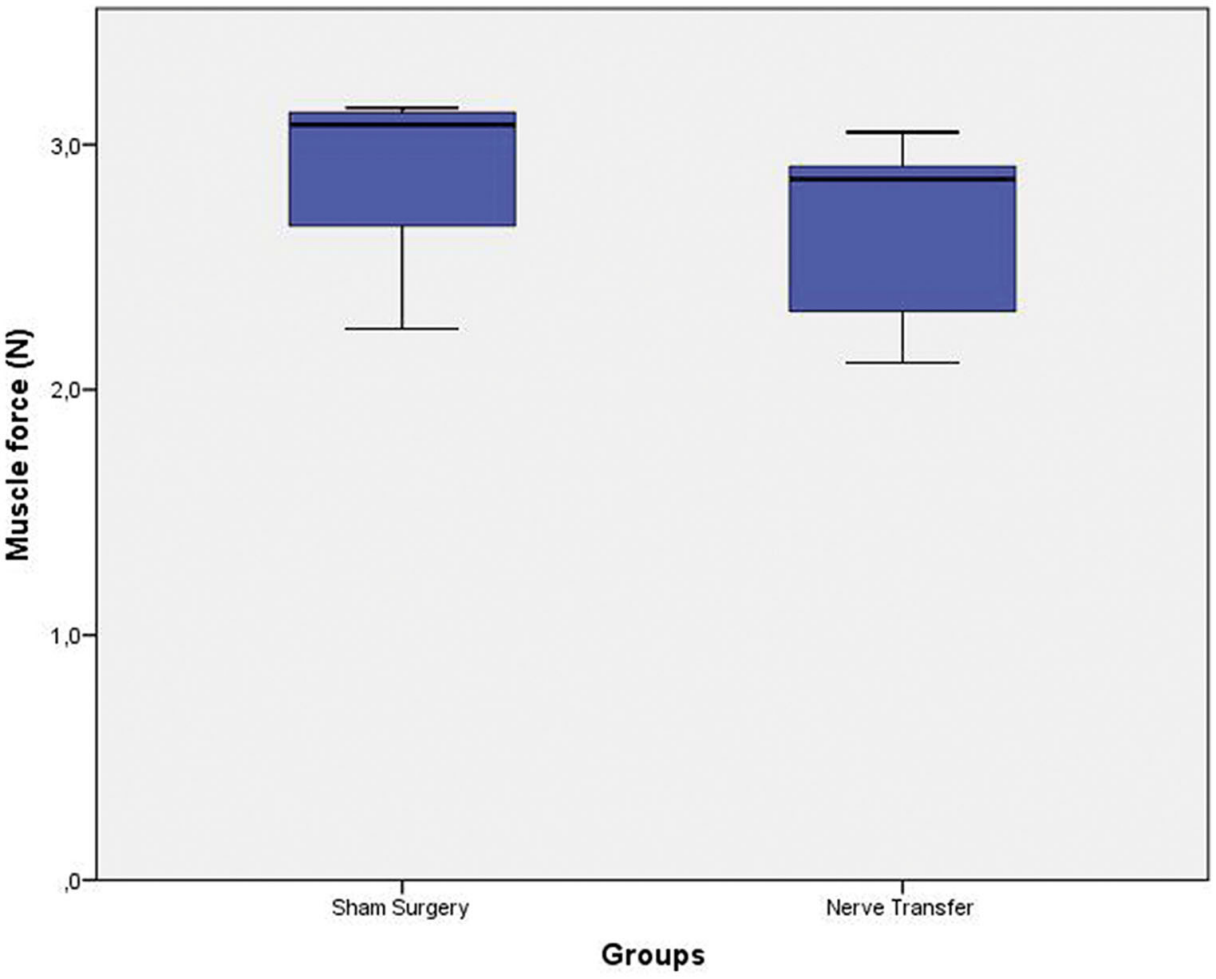
Muscle force regeneration: box plots of muscle force generated by the long head of the biceps, 12 weeks after sham or nerve transfer surgery. The sham surgery group had an average muscle force of 2.85 N ± 0.39 compared to 2.68 N ± 0.35 in the nerve transfer group. This difference was not statistically significant (*p* = 0.436)

Ten nerve transfer animals were allocated for muscle force analyses; however, one died during the protocol and was therefore excluded from the analyses. After 12 weeks of regeneration, the remaining nine nerve transfer animals achieved an average muscle force of 2.68 N ± 0.35 compared with 2.85 N ± 0.39 in five control animals (Fig. 3). This difference of 5.96 % was not statistically significant (*p* = 0.436). After the muscle force analyses, the ulnar nerve was cut and the musculocutaneus nerve stimulated which did not result in muscular contraction of the long head of the biceps.

### Behavioral testing

For the first 14 days after surgery the deficit after neurotomy of the musculocutaneous was evident, as the animals could not flex the elbow sufficiently. This deficit recovered gradually over the following weeks with full restoration of forelimb function at final analysis. At 12 weeks nerve transfer and sham animals were all able to reach behind the ears and thus scored the maximum of five points.

## Discussion

Nerve transfers are a powerful tool for reconstructive surgeons [23], but limited quantities of donor nerves often prevent better functional outcomes. Investigating the unknown neurophysiological effects of nerve transfers may therefore provide substantial knowledge to refine this technique and enable better functional outcome. Here we present an experimental nerve transfer model in the rat forelimb to investigate these effects in detail with standard neuroanatomical analyses.

For this purpose, we designed an experimental model based on clinical nerve transfer applications, such as standard elbow reanimation procedures [22, 24] and TMR [8, 10]. In this model we transferred the ulnar nerve directly onto the long head of the biceps after its original motor branch was divided. Thereby, a large donor nerve was available for reinnervation, and the denervation time of the muscle was limited to a minimum. This was considered important to focus the investigations on the nerve transfer and limit the effects of denervation on the results. After 12 weeks of regeneration, all nerve transfers had successfully reinnervated the targeted muscle, resulting in muscle force regeneration to 94.01 % compared with sham animals, which was not statistically different. No aberrant muscle reinnervation from the original musculocutaneus nerve was observed anatomically or functionally in any animals. The ulnar nerve was used as donor nerve because of similarity of clinical applications, easy access to surgical harvest, and minimal functional loss of the remaining forelimb. Operating time for the whole procedure was less than an hour and postoperative recovery unproblematic in all animals.

The forelimb was chosen for this model because 81 % of all nerve damages occur in the upper extremity and consequently so are nerve transfers [8, 15, 16, 22]. Although the principle of reconstructing nerves and muscle function is similar in the standard hindlimb model [12, 13] of neuromuscular research, differences in neuromuscular physiology and anatomy give reason to believe that the forelimb is a more suitable model to study nerve transfers’ neurophysiological effects [25–27]. This conclusion is based on differences between fore- and hindlimb in regard to muscle physiology, motor units, axon numbers, and different cortical representation [25, 28]. Previous studies have already shown that similar motor function between donor-to-recipient nerve and their axon count ratio plays a critical role in functional outcomes in humans and should therefore be closely matched in experimental model as well [16]. This is especially critical for the synergistic function between donor and recipient nerve to allow correct cortical recognition of the new innervation pattern by the patient [17, 29]. It has been shown that antagonistic nerve transfers lead to worse functioning of the targeted muscle and complicated rehabilitation processes with unsatisfying results [5, 18]. Neurophysiological analyses alone do not allow assessment of correct cortical reintegration into standard motion patterns. This, however, is of great importance in nerve transfers and feasible in the presented rat forelimb model.

**Fig. 4:**
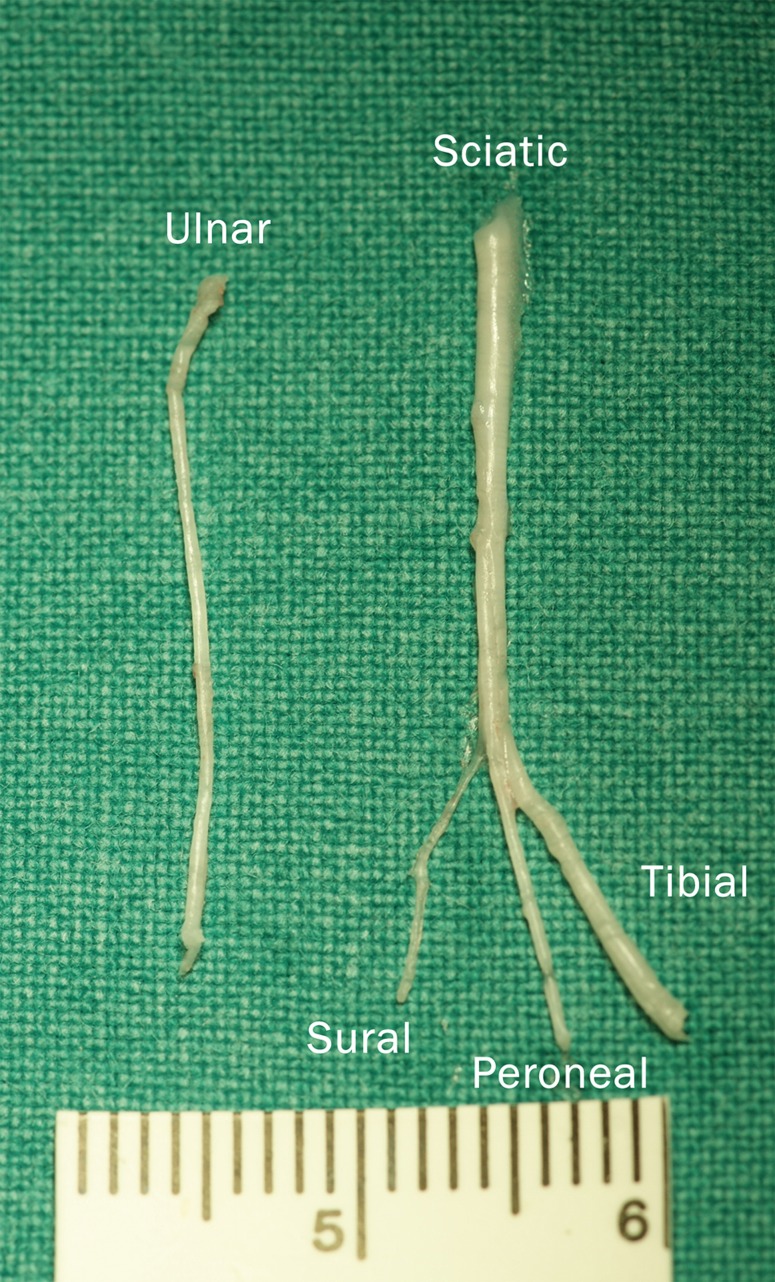
Anatomical differences in size: The ulnar and sciatic nerves were removed to illustrate the difference in size between forelimb and hindlimb model. The ulnar nerve is approximately one third of the sciatic nerve

In addition, multiple donor nerves (ulnar, median, radial, musculocutaneus, etc.) for nerve transfers with synergistic function and axon count ratios are available in this animal model [30, 31]. Furthermore, using the ulnar nerve as donor and the long head of the biceps as recipient led to minimal motor or sensory deficit and thus minimal animal burden. The ulnar nerve in the rat innervates the flexor carpi ulnaris, flexor digitorum profundus, interosseous muscles, lumbricals (four and five), and the abductor and flexor pollicis brevis [32]. After the nerve transfer, the animals showed deficits in elbow flexion due to the temporary denervation of the long head of the biceps and permanent deficits in paw pronation due to partial loss of intrinsic muscle function. However, none of these were observed to be critical for motor function, especially due to the rudimentary function of the pollex. Functional deficits were thus well compensated by the rats using functional synergistic muscles and could be monitored with the Bertelli test. Sensory deficits were only present at the lateral aspect of the fifth digit with small ulcers in only one animal. Although the ulnar nerve is responsible for sensory function of the medial forearm, digit four and five, a large part of this territory was probably resupplied by adjacent sensory nerves. As a consequence, no impact on routine activities such as food intake or climbing was observable. In comparison, the standard hindlimb model in neuromuscular research uses the sciatic nerve, which involves severe motor and sensory loss of function of the hindlimb. The resulting insensibility leads to severe auto-mutilation and may thus affect study results [12, 33]. From a methodological and animal welfare point of view, the forelimb may therefore present a refined model compared with previous standards, which have been previously reported [8, 28]. Limitations of this model include smaller anatomy of the forelimb and close proximity of the nerves to vital blood vessels, which may require additional microsurgical skills (Fig. 4). Additionally, the smaller and complex anatomy required an assistant in our study in order to retract muscles during the nerve transfer. On the contrary, many procedures can be performed by a single surgeon in the hindlimb model and may thus be easier to perform as there is no need for an assistant or an operating microscope with two binoculars.

Regarding standard neuromuscular analyses, we did not observe any restrictions in comparison with the hindlimb. In this study, we conducted muscle force and behavioral assessments. In addition, we successfully conducted muscle weight, intramuscular, and cut-axon retrograde labeling as well as high-density intramuscular electromyography (EMG) analyses, which are not reported here. To our knowledge, all standard analyses of neuromuscular research are applicable to this forelimb model with no restrictions.

In conclusion, this nerve transfer model in the rat forelimb presents a tool to investigate the neurophysiological effects of nerve transfers and subsequent changes in cognitive motor function. We could demonstrate that this model is reproducible, easy to apply and has a constant anatomy, and suitable for nerve transfers and their analyses with standard neuromuscular and behavioral tests.
